# The dual face of lamin A/C cardiomyopathy: risk prediction of heart failure and arrhythmias

**DOI:** 10.1093/eurheartj/ehag215

**Published:** 2026-05-13

**Authors:** Stephane Heymans

**Affiliations:** Department of Cardiology, Maastricht University, CARIM School for Cardiovascular Diseases, Universiteitssingel 50, Maastricht 6229 ER, The Netherlands; Centre for Molecular and Vascular Biology, Department of Cardiovascular Sciences, KU Leuven, Herestraat 49, Leuven 3000, Belgium; European Reference Network for Rare, Low Prevalence and Complex Diseases of the Heart (ERN GUARD-Heart)

## Abstract

**Graphical Abstract:**
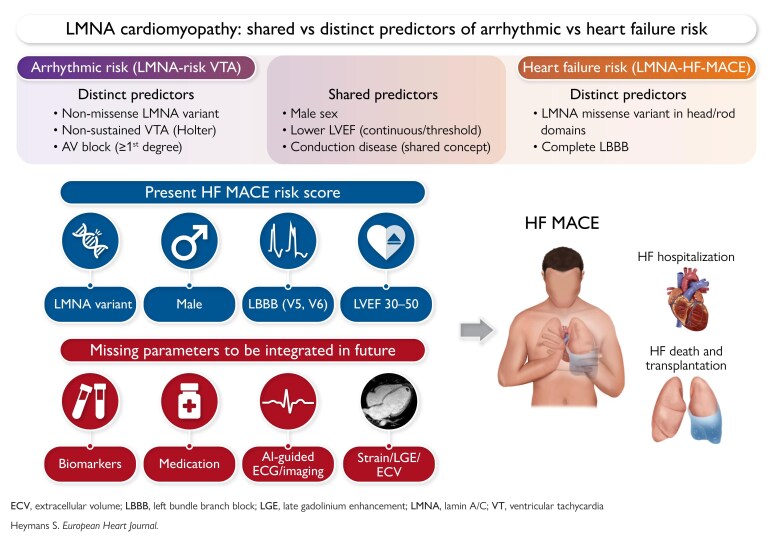

**This editorial refers to ‘Laminopathies: natural history and risk prediction of heart failure’, by P. Charron *et al.*, https://doi.org/10.1093/eurheartj/ehag104.**

Depending on the perspective and clinical focus, lamin A/C-related cardiomyopathy (LMNA-CMP) has traditionally been viewed either as a predominantly arrhythmogenic disorder or as a severe form of dilated cardiomyopathy (DCM). This apparent dichotomy reflects the true dual nature of laminopathies. The first disease-specific risk score, developed >5 years ago, focused on sudden cardiac death driven by ventricular tachyarrhythmias (VTA).^1^ This emphasis was justified: lamin A/C variants confer the highest risks of life-threatening VTA among all inherited cardiomyopathies, underpinning the strong rationale for early implantable cardioverter-defibrillator implantation.^2^ Yet the other face of an equally malignant trajectory of LMNA-CMP—progressive heart failure (HF)—has remained without a dedicated risk prediction tool.

The retrospective study by Charron and colleagues published in this issue of the *European Heart Journal*^3^ directly addresses this unmet need. Leveraging large multicentre retrospective cohorts in France with external international validation, the authors reposition HF from a late, inevitable complication to a clinically predictable outcome. They assess the 5-year risk of major heart failure events (HF-MACE). A combination of hard HF endpoints, including HF hospitalization, HF-related death, mechanical circulatory support, or heart transplantation, was used to study a risk score in 470 adults in France, followed by a successful validation in an international cohort. Four independent predictors of HF-MACE were identified: male sex, left ventricular ejection fraction (LVEF) <50%, missense variants in head and rod domains, and complete left bundle branch block (LBBB). A key strength relies in its simplicity and reliance on routinely available clinical variables. Importantly, predictive performance was preserved despite marked differences between derivation and validation cohorts, including disease stage, device use, and extracardiac involvement.

A biological and clinical comparison between the established LMNA VTA risk score and this new HF risk score is essential (*Graphical Abstract*). LMNA-CMP is a unified electrical–mechanical disorder, in which conduction abnormalities, malignant arrhythmias, cardiomyocyte death, and evolving cardiac failure occur in parallel and amplify each other.^4^ The HF-MACE used in this study are inherently heterogeneous: some components clearly reflect real HF progression, whereas others—such as heart transplantation or HF-related death—may overlap with the arrhythmogenic risk. It is therefore not surprising that both HF and VTA share key risk predictors: male sex, lower EF, and conduction disease. Distinct predictors also emerge. Nonsense LMNA variants, non-sustained VT, and atrioventricular (AV) block are associated with arrhythmic risk, whereas missense variants in head/rod domains and complete LBBB are distinct predictors of HF-MACE risk. This overlap underscores that VTA and HF are not competing outcomes but parallel manifestations of a shared myocardial substrate. Therefore, in clinics, both scores should be integrated and used for parallel prediction of HF and VTA risk, rather than in isolation.

Several limitations deserve consideration. First, patients with an LVEF <30% are excluded from the risk score, limiting its clinical applicability in this important high-risk subgroup. These patients exhibited a 1-year incidence of HF-MACE of 50% and had to be excluded due to violations of statistical assumptions. Their outcomes are strongly different: they had 5-year cumulative incidences of HF-MACE of 72%, and a cumulative incidence of advanced HF-MACE (AdV-MACE) of 22% within 1 year. These patients are precisely those in whom anticipatory decision-making—advanced HF referral, transplant evaluation, or clinical trial enrolment—may have the greatest impact. Their exclusion highlights that HF risk in LMNA-CMP evolves over time and may be better captured by stage-adapted or dynamic models rather than by a single static score. More work for international cohorts.

Second, predictive biomarkers (pro-B-type natriuretic peptide [proBNP], high sensitivity C-reactive protein [hsCRP], or hs-troponins) and advanced imaging parameters were not studied in the model, because of their missingness in both the test and validation cohorts. Biomarkers such as BNP or N-terminal proBNP (NT-proBNP) are particularly relevant in LMNA-CMP, reflecting myocardial stress, early dysfunction, and disease progression, often rising even in asymptomatic carriers or in those with mildly reduced EF.^5^ Higher levels consistently correlate with worse outcomes and have already been used as inclusion criteria in LMNA-CMP clinical trials.^6^ Similarly, cardiac magnetic resonance (CMR) imaging has become integral to baseline assessment in genetic DCM.^7,8^ Beyond late gadolinium enhancement (LGE), which predicts life-threatening arrhythmias and worse outcome,^9^ contemporary CMR imaging provides quantitative assessment of myocardial strain and extracellular volume as important risk predictors in LMNA-CMP.^10^ Impaired left atrial strain and LV global longitudinal strain predict adverse outcome in DCM patients to a degree comparable with LGE or EF.^11^ Future adaptations of the HF risk score should therefore explore/integrate the extra value of cardiac biomarkers and advance CMR imaging, moving from clinical surrogates towards mechanistic markers of myocardial stress and injury.

Third, both cohorts had a low use of recent HF therapies such as sogium–glucose co-transporter 2 (SGLT2) inhibitors and angiotensin receptor–neprilysin inhibitors (ARNIs), also reflecting—as for CMR imaging—the inclusion of older cohorts. Future validation of this score in more contemporary cohorts diagnosed after 2017 is warranted and should account for modern HF therapy, alongside biomarkers and advanced imaging.

The distinct types of LMNA variants involved in VTA (nonsense variants) and HF risk (missense variants in head/rod domains) are particularly intriguing, as the only pathophysiologically distinct factors in the HF/VTA risk scores. Missense LMNA variants lead to amino acid substitutions and typically exert dominant-negative effects, disrupting nuclear architecture and protein interactions in both fibroblasts and cardiomyocytes. This mechanism is associated with earlier electrical disease including AV block and ventricular arrhythmias, preceding systolic dysfunction.^12^ In contrast, nonsense LMNA variants cause a premature stop codon, leading to a truncated poisoned protein which may be degraded by nonsense-mediated decay. Haploinsufficiency, i.e. reduced levels of the wild-type normal protein, may result in a more direct dysfunction/suffering of cardiomyocytes, resulting in early LV dysfunction and HF progression.^13^ The incidence of LMNA-CMP patients with normal EF may be low in the current study, which could have led to under-representation of missense variants with high arrhythmic burden but preserved systolic function. As gene-specific therapies enter clinical development,^14^ variant type and location will probably prove crucial for interpreting therapeutic effects on arrhythmic vs HF outcomes.

Finally, further refinements/adaptations of the risk score could include paediatric patients, the impact of extra-cardiac involvement, and more granular stratification of patients with a normal EF but an arrhythmogenic phenotype. Achieving this will require more refined, machine learning-supported imaging and ECG, together with deep phenotyping.

In summary, this study represents an important step forward in the management of LMNA-CMP, extending the prevention of arrhythmia towards anticipation of HF progression. The key challenge is no longer whether HF risk can be predicted, but how such a prediction will be used to modify the natural history of a persistently progressive disease. As gene-(specific) therapy and RNA-based therapies enter the clinical arena, timely identification of patients on a trajectory towards HF may prove to be the most consequential form of risk stratification.

**Key messages**

- Four independent predictors of HF-MACE were identified in LMNA-CMP patients: male sex, LVEF <50%, missense variants in head and rod domains, and complete left bundle block.
- HF risk stratification will be central as gene-(specific) therapies emerge.
- Overlapping predictors—male sex, cardiac dysfunction, and conduction abnormalities—reflect shared biology and argue for integrated risk assessment.
- Exclusion of advanced disease stages (EF <30%) may limit its usability and calls for functional stage-adapted approaches.
- Future validation in more recent cohorts has to include CMR measures (LGE, extracellular volume, and strain, among others) and relevant biomarkers.
